# Noninvasive Ultrasound Imaging in Juvenile Idiopathic Arthritis: Diagnostic and Findings on the Temporomandibular Joint—A Prospective Study

**DOI:** 10.1155/ijod/9491663

**Published:** 2025-07-29

**Authors:** Marco Farronato, Paolo Cressoni, Davide Farronato, Giovanni Cattaneo, Irene Borzani, Roberto Biagi, Cinzia Maspero

**Affiliations:** ^1^Department of Biomedical Surgical and Dental Sciences, University of Milan, Milan, Italy; ^2^Department of Biomedical Surgical and Dental Sciences, Fondazione IRCCS Cà Granda Ospedale Maggiore Policlinico, Milan, Italy; ^3^Department of Medicine and Surgery - School of Dentistry, University of Insubria, Varese, Italy; ^4^Department of Medicine and Surgery, University of Milan Bicocca, Milan, Italy; ^5^Department of Radiology, Pediatric Division, Fondazione IRCCS Cà Granda Ospedale Maggiore Policlinico, Milan, Italy

**Keywords:** diagnosis, juvenile idiopathic arthritis, temporomandibular joint, ultrasound

## Abstract

**Introduction:** Juvenile idiopathic arthritis (JIA) is a chronic autoimmune condition. The temporomandibular joint (TMJ) is one of the most affected joints in JIA. It can bring significant symptoms and impairments if not treated, and routinely instrumental exams are necessary to track its progress during the visits. The purpose of this study was to determine the efficiency in tracking the status of TMJ involvement with ultrasound (US) imaging in patients with a diagnosis of JIA and to assess its effectiveness in detecting different alterations.

**Materials and Methods:** Inclusion criteria included patients previously diagnosed with JIA to be recruited in this prospective observational study. Each patient underwent detailed US evaluation of the TMJ to assess for various pathological changes, including condylar profile alterations, erosive phenomena, bone apposition, osteophyte formation, disc displacement, and soft tissue changes. The assessment was performed by two expert blinded operators. The US findings were compared with clinical manifestations and conventional imaging, for sensitivity, specificity, and predictive values.

**Results:** A total of 46 patients divided into 39 female and 7 males, between 7 and 19 years were recruited. Of the recruited patients, 15% showed discordance and were asymptomatic, while 85% of the patients showed at least one joint manifestations. Sensitivity, specificity, and negative predictive value (NPV) of US for detecting TMJ pathology were calculated using conventional imaging as the reference standard.

**Conclusions:** US showed a good concordance with traditional diagnosis, however it does not substitute traditional imaging for diagnosis. US demonstrated potential to be a reference noninvasive tool for monitoring TMJ secondary lesions in JIA and for monitoring during routine visits, offering advantages, such as noninvasiveness, cost-effectiveness, and real-time dynamic imaging capabilities.

## 1. Introduction

Juvenile idiopathic arthritis (JIA) is a group of different chronic inflammatory joint diseases affecting primarily children and adolescents. Its prevalence is estimated to be between 16 and 150 per 100,000 children [1]. These conditions are characterized by joint arthritis lasting at least 6 weeks with idiopathic origin in children under the age of 16 years. JIA can affect various joints, especially the temporomandibular joint (TMJ) is a commonly affected site. The involvement of TMJ was observed in more than 30% of patients with a diagnosis of JIA after the age of 18 [2]. TMJ arthritis in JIA can lead to pain, restricted jaw movements, and growth alterations of the mandible, a primary ossification center. The asymmetrical growth pattern can potentially alter facial esthetics and overall quality of life with limited functions, if not treated [3, 4 ].

The pathogenesis of TMJ involvement in JIA is multifactorial, involving immune inflammation and autoimmune response, synovial hyperplasia, and alterations in the condylar cartilage and subchondral bone [5]. The early detection of TMJ arthritis is mandatory to prevent complications, such as micrognathia or II class tendency. Also, impaired craniofacial growth on the lower third of the face in long-term might occur [6–8]. This often requires regular monitoring that has to be accessible and repeatable with low costs in the daily practice. The conventional imaging systems suggested by the international guidelines are the magnetic resonance imaging (MRI) and the computed tomography (CT). Those are often used to evaluate TMJ pathology but may have limitations, for example in the costs and accessibility of the MRI, and radiation exposure of the CBCT, especially in pediatric populations [9].

Ultrasound (US) has been described by various authors as a promising alternative for analyzing the TMJ involvement due to its noninvasive nature. The lack of ionizing radiation, cost-effectiveness, and real-time dynamic assessment of joint structures potentially makes it a very effective tool [10–15]. US allows the evaluation of both static and dynamic parameters of the TMJ, including: condylar morphology, erosive changes, disc displacement, and soft tissue abnormalities, such as thickening of the joint capsule and periarticular tissues [16, 17]. US, in general, could offer a balance between sensitivity, noninvasiveness, and practicality. Despite these advantages, the use of US is not yet standardized and is operator dependent [18, 19]. This study aims to evaluate the concordance between the US and MRI, and to analyze the findings retrievable from US imaging for routine checks in patients with JIA. We hypothesize that US can provide detailed information for the longitudinal assessment of TMJ involvement, improving outcomes for pediatric and young adult patients with JIA.

## 2. Materials and Methods

This prospective blinded observational study aimed to evaluate US in detecting TMJ involvement in patients diagnosed with JIA. The study was conducted at University of Milan, Ospedale Policlinico Fondazione IRCCS Cà Granda Milan, Italy, between 2020 and 2024. Ethical approval was obtained from the Institutional Review Board (IRB) N. 3. UOC 420/425. 1/9/2018, and informed consent was obtained from all participants or their legal tutors. Due to the noninvasiveness of the exams all the patients considered eligible were recruited.

### 2.1. Patient Selection

A total of 46 patients diagnosed with oligoarticular and poliarticular JIA, aged between 7 and 19 years with an onset of the illness before the age of 14, were recruited from the pediatric rheumatology clinic at IRCCS Cà Granda Ospedale Policlinico. Inclusion criteria included a confirmed diagnosis of JIA according to the International League of Associations for Rheumatology (ILAR) classification criteria and clinical manifestations of TMJ involvement based on symptoms, such as pain, restricted jaw movement, and joint swelling by the rheumatology department from our institution [20 –22]. Patients with contraindications to US or inability to cooperate with the procedure were excluded from the study. Other exclusion criteria were applied, such as past jaw or maxillo-facial interventions, history of craniofacial trauma, systemic disease or syndromes, active orthodontic, or orthopedic treatment. Also patients with a late onset after the age of 14 were excluded to uniform the sample.

### 2.2. US Imaging Protocol

All participants underwent a detailed US evaluation of both TMJs using a high-frequency linear array transducer (frequency range, 12–18 MHz) and a standardized imaging protocol. The US examinations were performed by two experienced radiologists specialized in musculoskeletal US, who were blinded to the patients' clinical and conventional imaging findings.

The US protocol included both static and dynamic assessments of the TMJ, focusing on the following parameters:

- Condylar morphology: Evaluation of condylar shape (regular vs. irregular), contour abnormalities, and presence of erosive changes.
- Disc position: Assessment of disc position (normal vs. displaced) during mouth opening and closing maneuvers.
- Soft tissue changes: Measurement of joint capsule thickness, assessment for periarticular soft tissue swelling.
- Dynamic function: Real-time assessment of condylar movement during active mouth opening to evaluate for range of motion abnormalities and condylar excursion. During dynamic assessment, movements, such as lateral excursions and protrusion were also evaluated to analyze the functional joint mechanics in real-time.

### 2.3. Image Analysis and Interpretation

US images were digitally stored and independently reviewed by the two radiologists with expertise in musculoskeletal imaging. Discrepancies in image interpretation were resolved through discussion until agreement was reached. Quantitative measurements of condylar dimensions, disc position, and soft tissue changes were recorded using electronic calipers integrated into the US machine software. A total of 12 records were obtained for each patients and divided for each condyle or bilaterally.

### 2.4. Statistical Analysis

All statistical analyses were conducted using Microsoft Excel (Microsoft, WA, USA) and R software (version 4.0.3). Descriptive statistics were used to summarize the demographic characteristics, clinical features, and US findings of the study population. Continuous variables were reported as means with standard deviations (SDs) or medians with interquartile ranges (IQRs), depending on the distribution. Categorical variables, such as the presence of TMJ abnormalities were expressed as frequencies and percentages. *p*-Values <0.05 were considered statistically significant. Correlation between US findings and clinical symptoms was assessed using Pearson's chi-squared test for categorical variables and Spearman's rank correlation for continuous variables.

To evaluate the diagnostic accuracy of US in detecting TMJ pathology, sensitivity, specificity, positive predictive value (PPV), and negative predictive value (NPV) were calculated, with MRI used as the reference standard.

### 2.5. Sample Size Calculation for the Study

A priori sample size calculation was conducted based on the anticipated sensitivity and specificity of US imaging in detecting TMJ pathology in patients with JIA. The sample size calculation was based on the primary outcome of the diagnostic accuracy of USs compared to the gold standard, magnetic resonance.

The power of the study was set at 80% (*β* = 0.20) to detect a clinically significant difference, with a two-tailed significance level (*α*) set at 0.05. For the calculation, a previous study by Müller et al. [23 ] was chosen.

Thus, a minimum of ~62 TMJ joints (or 31 patients) would be required to achieve the desired power and precision. Considering potential dropouts or incomplete data, a target enrollment of 70 TMJ joints was set. Ultimately, 46 patients (92 TMJ joints) were included in the study.

## 3. Results

### 3.1. Demographic and Clinical Data Analysis

The demographic data of the study participants were:

  Mean age: 15.4 years (SD = 3.4 years)
  Gender distribution: 39 females (84.78%) and 7 males (15.22%)

A total of 46 patients diagnosed with JIA were included, comprising (84.78%) and 7 males (15.22%). The mean age of the participants was 15.4 years (SD ± 3.4, range 7–19 years).

### 3.2. US Features of TMJ Involvement

#### 3.2.1. Condylar Morphology

US revealed structural abnormalities, both unilateral or bilateral, in the TMJ condyles in 26 patients (56.52%). “Irregular morphology” was defined as any alteration in the smooth contour of the condyle, including surface undulations or loss of the typical convex profile. Flattening, defined as a reduction in the expected convexity of the condylar head, was found in 16 patients (34.78%). Condylar prominence, indicating a more protruded bony contour compared to the contralateral or expected morphology, was present in 13 patients (28.26%). Erosive changes or fissures were found in four patients (8.70%) that includes cortical discontinuity or hypoechoic defects along the condylar surface.

#### 3.2.2. Disc Position and Movement

During real-time dynamic US 18 patients (39.13%) demonstrated restricted condylar excursions. Restricted movement was defined as reduced anteroposterior displacement of the condyle during mouth opening, with or without evident asymmetry. These dynamic evaluations indicated either unilateral or bilateral involvement, associated with subjective complaints of restricted mouth opening.

#### 3.2.3. Soft Tissue Changes and Vascular Findings

Soft tissue abnormalities were identified in 10 patients (21.74%). These characterized by thickened joint capsules (>0.1 cm), consistent with synovial inflammation. Additionally, three patients (6.52%) showed articular incongruence or joint effusion, defined as loss of parallelism between the articular surfaces. One patient (2.17%) exhibited osteophytes formation, reduced capsule thickness, and hypervascularity on power Doppler, suggestive of active inflammation.

**Figure 1:**
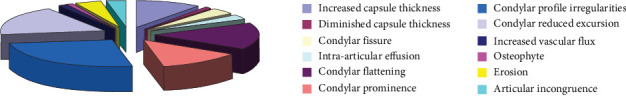
The diagram of the findings.

**Figure 2:**
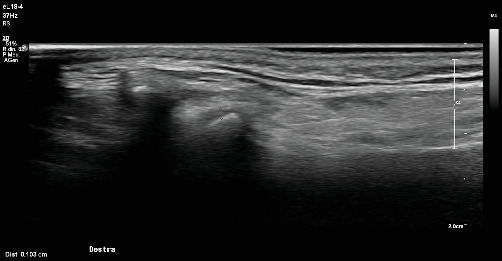
TMJ ultrasound: Erosion and flattening of the condylar head in RA—coronal scan, the flattening is visible between the two pointers in the viewer.

**Figure 3:**
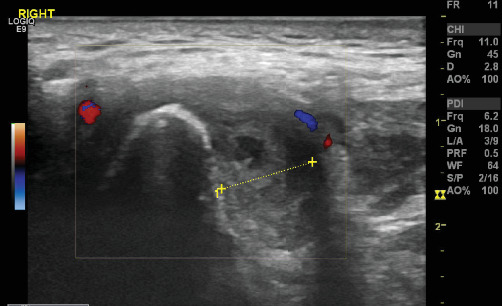
TMJ ultrasound: Intra articular effusion with synovial thickening, the yellow dotted line indicates the periarticular space width; the red and blue doppler signals indicate increased synovial vascularization, which is a sign of inflammation, the bony contour of the condyle looks irregular with signs of erosion.

In total, 39 patients (84.80%) presented at least one sonographic alteration, while seven patients (15.20%) showed normal bilateral TMJs (Tables 1 and 2) (Figures 1–3).

#### 3.2.4. Diagnostic Accuracy Comparison With Conventional Imaging

Direct comparison of US findings with conventional imaging modalities (MRI) was feasible in the 30 patients having at least another recent MRI taken. On these patients US demonstrated a sensitivity of 85% and specificity of 80% for detecting TMJ pathology when compared with MRI findings, which is the gold standard. PPV and NPV were calculated at 88% and 75%, respectively.

Statistical analysis revealed significant correlations between US findings and clinical symptoms. Pain on palpation and restricted jaw movement showed a statistically significant correlation respectively (*χ*^2^ = 10.25, *p*  < 0.01) and (*χ*^2^ = 7.85, *p*  < 0.05). The statistical analysis supports the findings with a significant correlation between US-detected TMJ defects and clinical symptoms in patients with JIA (Table 2).

## 4. Discussion

The findings of this study underscore the utility of US as an imaging modality for assessing TMJ involvement in pediatric patients affected by JIA [1, 3, 7 ]. US offers several advantages over conventional imaging techniques, including real-time dynamic assessment of joint morphology and function without ionizing radiation exposure. This is essential in the pediatric population, especially where repeated imaging may be necessary to monitor disease progression and treatment response during the growth stages of the patients. The ability of US to detect soft tissue abnormalities, such as joint capsule thickening, is particularly helpful due to the inflammatory nature of JIA and hints that US can be used as an early detection system for joint involvement before structural changes may occur [16]. The good concordance found with US in detecting TMJ pathology, as demonstrated in our study, highlights its potential role in early diagnosis and management of JIA-related TMJ involvement. These results are consistent with previous literature suggesting that, while US may not replace MRI in visualizing intra-articular structures, such as the disc, it holds significant promise in identifying condylar erosions, soft tissue thickening, and synovial inflammation [23, 24 ]. The diagnostic accuracy of US in our cohort in sensitivity (85%) and specificity (80%) suggests that US could be integrated into routine monitoring strategies, especially in cases where rapid serial illness onset is present. While MRI remains the gold standard, especially for detailed soft tissue evaluation, early detection allows for timely intervention and targeted treatment strategies aimed at mitigating disease progression and improving long-term outcomes, including preservation of joint function and reduction of pain. The detection of changes, especially subtle changes in asymptomatic patients, should give clinicians an indication on the clinical management of the patients. For example, it could give indications on more frequent clinical controls, or even to vary the drug prescription. However, to date there are no protocols on close follow-up without evidence of pain or other signs and it is important to add more frequent data collection with US and physical inspection. This summed with cross-checks of the clinical symptomatology might help to identify the cause or the signals of challenging symptoms or worsening of the disease. In general a richer data flow would be useful both for the daily practice and for data collection and follow-up of the asymptomatic patients reinforcing the role of US as a monitoring tool rather than a one-time diagnostic snapshot.

Future advancements in US technology, including higher resolution imaging and improved software for automated measurement by the use of artificial intelligence, could further enhance the overall diagnostic accuracy reducing the operator-dependent errors [25, 26].

## 5. Limitations

Despite its advantages, US is operator-dependent and requires specialized training for accurate interpretation of TMJ findings which may limit its widespread adoption. Variability in equipment quality and patient cooperation during dynamic maneuvers may also affect diagnostic accuracy. Moreover, while US offers insights into morphological and vascular changes, its ability to visualize intra-articular structures, such as the articular disc is limited compared with MRI.

Future research may focus on refining US protocols to enhance sensitivity and specificity in detecting early TMJ changes associated with JIA or including the use of Artificial Intelligence. Longitudinal studies are needed to evaluate the prognostic value of US findings and their correlation with clinical outcomes over time. In order to avoid selection bias a more consistent sample is needed. Additionally, comparative studies exploring the cost-effectiveness of US versus MRI in pediatric rheumatology practice would provide valuable insights into healthcare resource allocation.

## 6. Conclusion

In conclusion, US emerges as a promising adjunctive tool for assessing TMJ involvement in pediatric patients with JIA, offering real-time dynamic assessment of joint morphology and function. The findings of this study support its integration into routine clinical practice for early detection and monitoring of TMJ pathology, also facilitating timely intervention and improved outcomes for affected individuals.

## Figures and Tables

**Table 1:** Prevalence of the ultrasonic findings.

Diagram	Legends	
Condylar irregularities	26	56.52%
Condylar flattening	16	34.78%
Condylar prominence	13	28.26%
Erosive changes	4	8.70%
Restricted condylar excursion	18	39.13%
Soft tissue abnormalities	10	21.74%
Joint effusion	3	6.52%
Osteophytes or reduced capsule thickness	1	2.17%
Patients with no alterations	7	15.20%

**Table 2:** Right left and bilateral findings.

TMJ side	None	Right	Left	Bilateral
Increased capsule thickness	36	2	4	4
Diminished capsule thickness	45	0	1	0
Condyle fissure	42	1	3	0
Intra-articular effusion	43	1	2	0
Condylar flattening	30	3	8	5
Condylar prominence	33	6	7	0
Condylar profile irregularities	20	7	9	10
Condylar reduced excursion	28	5	11	2
Increased vascular flux	45	0	0	1
Osteophyte	45	0	0	1
Erosion	42	1	3	0
Articular incongruence	43	0	3	0

## Data Availability

The authors will make additional data available upon reasonable request. The data that support the findings of this study are available from the corresponding author upon reasonable request.
